# The Effect of Service on Microstructure and Mechanical Properties of HR3C Heat-Resistant Austenitic Stainless Steel

**DOI:** 10.3390/ma13061297

**Published:** 2020-03-13

**Authors:** Grzegorz Golański, Adam Zieliński, Marek Sroka, Jacek Słania

**Affiliations:** 1 Czestochowa University of Technology, Department of Materials Engineering, Armii Krajowej 19, 42-200 Częstochowa, Poland; grzegorz.golanski@pcz.pl; 2 Łukasiewicz Research Network - Institute for Ferrous Metallurgy, K. Miarki 12-14, 44-100 Gliwice, Poland; azielinski@imz.pl; 3 Department of Engineering Materials and Biomaterials, Silesian University of Technology, Konarskiego St. 18a, 44-100 Gliwice, Poland; 4 Faculty of Mechanical Engineering and Computer Science, Czestochowa University of Technology, Armii Krajowej 21, 42-200 Częstochowa, Poland; jacek_slania@poczta.onet.pl

**Keywords:** HR3C steel, precipitates, mechanical properties, creep resistance

## Abstract

The physical metallurgical tests were performed on the test samples made of HR3C steel, taken from a section of a pipeline in the as-received condition and after approximately 26,000 h of service at 550 °C. In the as-received condition, the test material had austenitic microstructure with numerous large primary Z-phase precipitates inside the grains. The service of the test steel mainly contributed to the precipitation processes inside the grains and at the grain boundaries. After service, the following precipitates were identified in the microstructure of the test steel: Z-phase (NbCrN) and M_23_C_6_ carbides. The Z-phase precipitates were observed inside the grains, whereas M_23_C_6_ carbides - at the boundaries where they formed the so-called continuous grid. The service of the test steel contributed to the growth of the strength properties, determined both at room and elevated temperature (550, 600 °C), compared to the as-received condition. Moreover, the creep properties of HR3C steel after service were higher than those of the material in the as-received condition. The increase in the strength properties and creep resistance was connected with the growth of strengthening of the test steel by the precipitation of Z-phase and M_23_C_6_ carbides.

## 1. Introduction

The HR3C steel belongs to a modern group of creep-resistant austenitic steels. This steel was developed as a result of modification of the chemical composition of steel 310 by adding the niobium and nitrogen microadditions and by optimizing the nickel content, which resulted in the growth of high-temperature creep strength and creep resistance while maintaining very high heat resistance. High creep resistance and very good resistance to high-temperature corrosion and oxidation in steam make the HR3C steel the recommended material for the elements of supercritical power units, such as, but not limited to, boiler superheaters working at 650–660 °C [1,2].

The laboratory tests of HR3C steel made so far confirmed the assumed high corrosion resistance, but at the same time showed its susceptibility to very fast decrease in impact strength, with the cracking mechanism changed from ductile to brittle-intercrystalline [3,4,5,6,7]. The performed tests showed what was responsible for the low impact strength of HR3C steel after ageing at above 600 °C. It was the unfavourable morphology of the secondary phases—M_23_C_6_ carbides—precipitated at the grain boundaries, as well as the coarse-grained structure of the steel. Quick loss of ductility of HR3C steel indicates that the recommendation of the steel as the material for the boiler superheater pipes requires a certain amount of providence [4,6,7]. However, there is a lack of data concerning the HR3C steel behaviour during the actual service in the power unit. This paper presents the results of research on the structure and mechanical properties of the test samples taken from a section of the boiler superheater pipe made of HR3C steel after service at 550 °C.

## 2. Material and Methodology of Research

The tests were carried out on 25Cr – 20Ni – Nb – N (HR3C) creep-resistant austenitic steel. The tests samples were taken from a section of the steam superheater of 45 mm by 5.7 mm. The tests were carried out on the material in the as-received condition and after approximately 26,000 h service at 550 °C. The chemical composition of the test steel was determined using the SpectroLab spark spectrometer and are presented in Table 1.

The microscopic tests were performed on metallographic specimens etched with the Mi19Fe reagent, using the scanning electron microscope Jeol 6610LV (SEM) compatible with the chemical composition analyser X-Max Oxford Instruments (EDS). The metallographic specimens were taken perpendicularly to the axis of the pipe section. The analysis of precipitates was performed with the transmission electron microscope TITAN 80–300 using the selected area electron diffraction with thin foils. The 3 mm discs with thickness of 100–120 μm were electrolytically thinned to perforation using a solution of 20% perchloric acid in ethanol at −30 °C and the applied voltage of 20 V. The scope of the tests of mechanical properties included: Static tensile tests at room temperature and elevated temperature (550, 600 °C) using flat test pieces with the original gauge width of b_o_ = 10 mm—the testing machine Zwick/Roell Z250, and the accelerated creep tests at the test stress of 100 MPa and temperatures of: 700, 720, 740, 760, and 780 °C. The detailed description of the accelerated creep test methodology is presented elsewhere, inter alia in [8].

## 3. Test Results and Their Analysis

**Figure 1:**
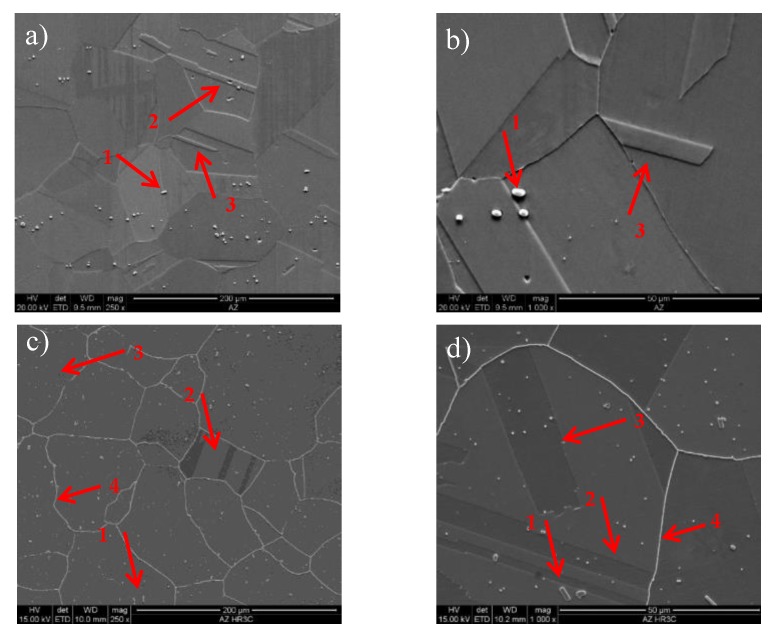
(**a**,**b**) microstructure of HR3C in the as-received condition, (**c**,**d**) microstructure of HR3C after service, 1—primary precipitates, 2—coherent twin, 3—incoherent twin, and 4—precipitates at the grain boundary; SEM.

In the as-received condition, the HR3C steel was characterised by a coarse-grained austenitic microstructure with irregular shape and large primary precipitates (Figure 1a,b). The vast majority of Z-phase particles were randomly distributed in the grains and a small amount was observed at the grain boundaries. Some of the particles in the test alloy were arranged as bands of precipitates.

**Figure 2:**
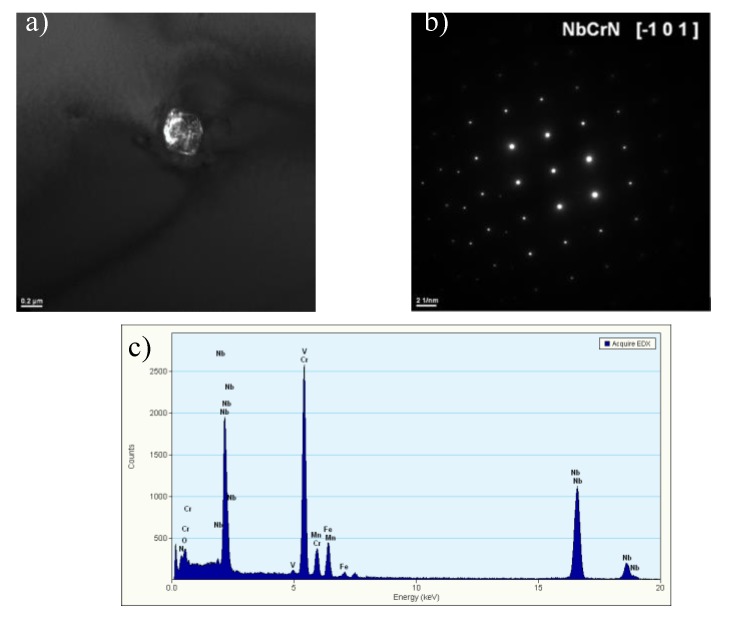
Primary Z-phase precipitate (**a**), resolved diffraction (**b**), EDS analysis (**c**); TEM.

These particles were identified as undissolved primary Z-phase. The typical morphology of the primary Z-phase is showed in Figure 2. As residual particles, these precipitates were formed during solidification and are undissolved by the solution treatment [9,10]. The Z-phase in the as-received HR3C steel was also observed by Zieliński [3], while Bai [6] and Peng [11] reported the presence of primary Nb-rich MX particles in the as-received samples. The presence of primary precipitates in austenitic steels stabilised by microadditions of niobium or/and titanium is a typical feature of these materials. The primary precipitates of Z-phase are unfavourable precipitates in the microstructure of creep-resisting austenitic steels, since they do not have an influence on the precipitation strengthening due to their size. They can be places of potential nucleation and growth of creep cavities at the interphase precipitate/matrix boundary and block the precipitation of nanoscale Nb-rich phase during creep/service [12]. In austenitic steels, the role of primary precipitates is limited to binding carbon atoms or/and nitrogen and impeding the grain growth during the thermo-plastic treatment. According to [13,14], the primary precipitates in austenitic alloys may be transformed to harmful TCP (topologically close-packed) type phase(s) during service. Additionally, a number of annealing twins in the grains are observed. The twin boundaries in the austenite steel could improve the plasticity and ductility by interrupting the continuity of the austenite grain boundaries and relaxing the stress at the austenite grain boundaries [4,6]. According to [15], two types of twin substructures, coherent twins and incoherent twins are observed in HR3C steel. The coherent twins have straight sub-grain boundaries across the entire austenite, while incoherent twins are inside the austenite grain as shown in Figure 1, arrows two and three, respectively. No precipitates were observed at the grain boundaries and the twin boundaries.

**Figure 3:**
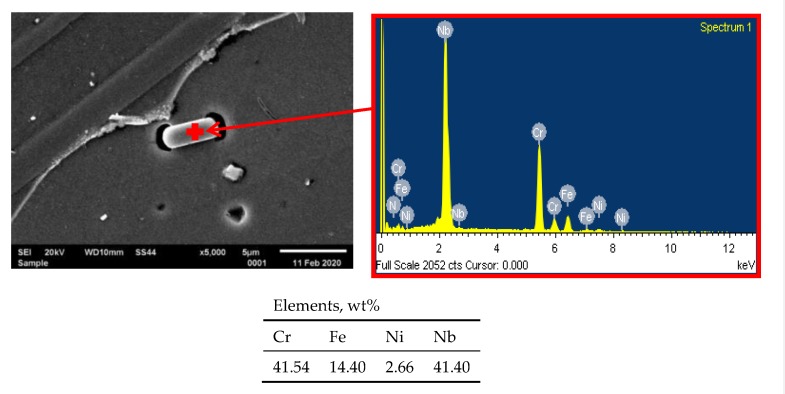
EDS analysis of precipitate inside the grain.

**Figure 4:**
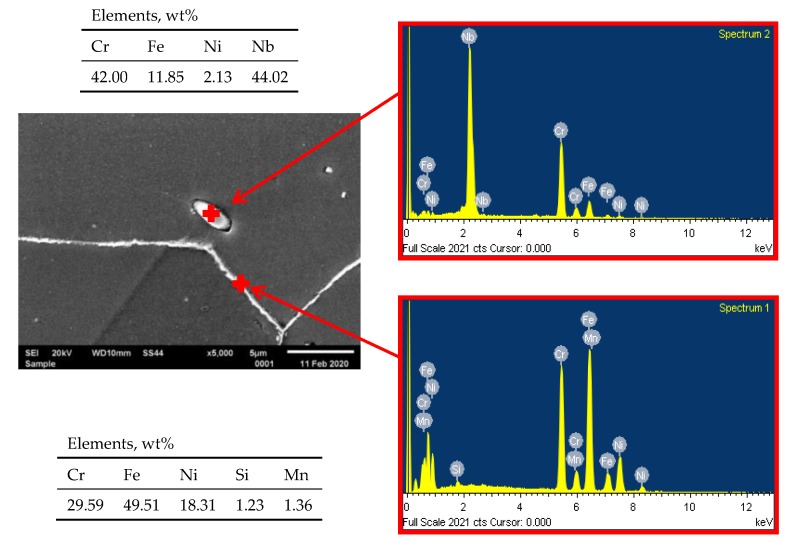
EDS analysis of precipitate inside the grain and at the grain boundary.

The microstructure of HR3C steel after service is presented in Figure 1c,d. The results show that the service, compared to the as-received condition (Figure 1a,b), induced the excessive precipitation especially at the grain boundaries, and also inside the grains. Inside the grains, relatively numerous precipitates of varying size are visible, whereas at the grain boundaries the particles form the so-called continuous grid of precipitates (Figure 1c,d). The results of EDS analysis show that the precipitates at the grain boundaries are rich in chromium, however, inside the grains they are rich in chromium and niobium (Figure 3 and Figure 4).

**Figure 5:**
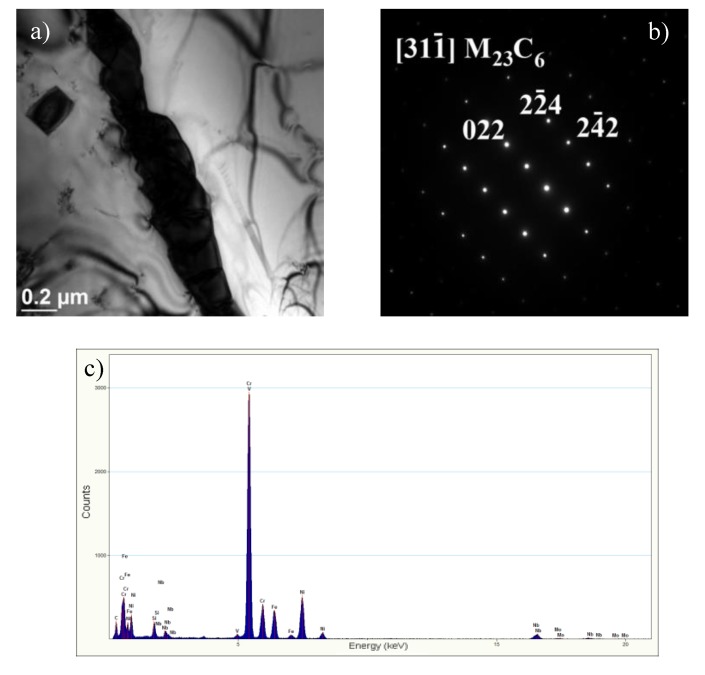
M_23_C_6_ carbides at the grain boundaries (**a**), resolved diffraction (**b**), EDS analysis (**c**); TEM.

**Figure 6:**
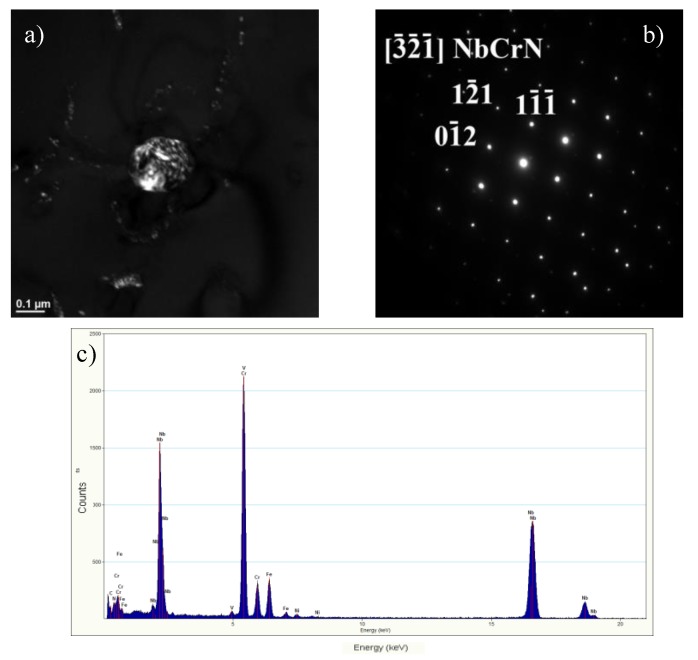
Secondary Z-phase (**a**), resolved diffraction (**b**), EDS analysis (**c**); TEM.

At the grain boundaries, these precipitates were identified as Cr-rich M_23_C_6_ carbides (Figure 5), whereas inside the grains, as the secondary precipitates of Nb- and Cr-rich Z-phase (Figure 6). In the alloys, like in the steels, the preferential place of precipitation of particles is the grain boundaries. As surface defects, the grain boundaries are characterised by higher interfacial energy compared to that inside the grain and at the twin boundaries. As defects with a disordered structure, the grain boundaries are also the areas that enhance faster diffusion of alloying atoms compared to the grain interior [10,16,17,18]. This allows for the preferred precipitation of M_23_C_6_ carbides at the grain boundaries. The M_23_C_6_ carbide precipitation process in austenitic steels is related to the reduction of carbon solubility with temperature [10]. Moreover, the precipitation of M_23_C_6_ carbides is a process of redistribution and reduction of the strain energy [6]. The M_23_C_6_ carbides precipitated at the grain boundaries in the austenitic steel can be observed as early as after around 1-h creeping at 600 °C [10,16]. The fine M_23_C_6_ particles precipitated at the grain boundaries increase the creep resistance of austenitic steels by impeding the grain boundary sliding [9,10]. Prat et al. [19] found that the formation of nanosized M_23_C_6_ carbides could provide good creep resistance due to pinning effects of grain boundaries. The positive influence of this interaction depends on the stability of the particles precipitated at the boundaries and disappears with the growth of the particles.

**Figure 7:**
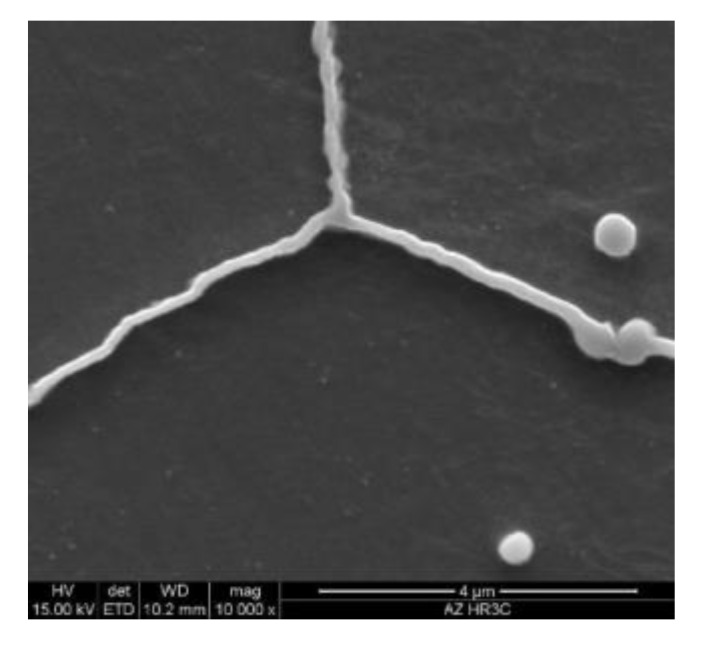
Widening of grain boundaries in HR3C steel after service.

However, M_23_C_6_ carbides are characterised by low thermodynamic stability [10,15,19,20], and this contributes to an increase in both their amount and size at the boundaries, which results in the loss of this favourable impact. This rapid growth and coarsening of M_23_C_6_ carbides greatly widened the grain boundaries (Figure 7). The coarsening of M_23_C_6_ carbides at the grain boundaries is very harmful to the properties of the materials—increase in embrittlement and loss of the strengthening effect of particles [6,11]. The precipitation and growth of M_23_C_6_ carbides, Cr-rich particles, at the grain boundaries may lead to the formation of chromium-depleted region near the grain boundaries [17,18]. When the content of chromium drops to the limit values needed for passivation, the intergranular corrosion will occur. Yan [21] suggests that the formation of depletion zone near the grain boundaries may affect a decrease in the local strength and promotes inhomogeneous deformation. 

Apart from the grain boundaries, the M_23_C_6_ particles can also gradually precipitate at the incoherent twin boundaries, coherent twin boundaries, and inside the grains. In the test steel after service, these precipitates were not observed at the twin boundaries (Figure 1c,d). The twin boundaries are characterised by low energy (3%–10% energy of the large-angle grain boundary), which results from the fact that the arrangement of atoms at both sides of the twin boundary is symmetric. Hence, the driving force for nucleation and growth of precipitates along the twin boundaries is lower than for the austenite grain boundaries [4]. The M_23_C_6_ precipitates were also not revealed inside the grains, which probably results from the relatively short time of service of the test steel.

**Figure 8:**
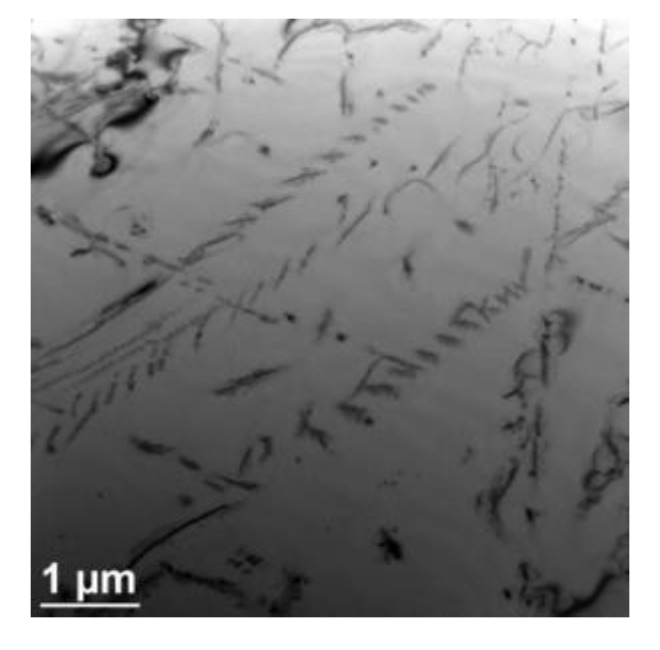
Z-phase aggregation precipitates inside the austenite grain.

Inside the grains, the secondary dispersive Z-phase precipitations were observed in the steel (Figure 6 and Figure 8). The Z-phase particles can precipitate independently by the in situ mechanism or as a result of the local conversion of MX precipitates into the Z-phase, probably by a mechanism similar to the formation of this phase in 9%–12%Cr martensitic steels [22,23]. The Z-particles precipitate near the grain boundaries and also on the dislocations. Similarly, the precipitation of secondary Z-phase particles occurs in areas with high dislocation density, which results not only in the pile-up of dislocations, but also in the aggregation of precipitates (Figure 8). According to the Orowan law, the fine-dispersion Z-phase precipitates and their relatively high stability make the particles/aggregates effectively prevent the motion of dislocations and have an intensive pinning strengthening effect [10,11,23,24].

## 4. Mechanical Properties of HR3C Steel after Service

**Figure 9:**
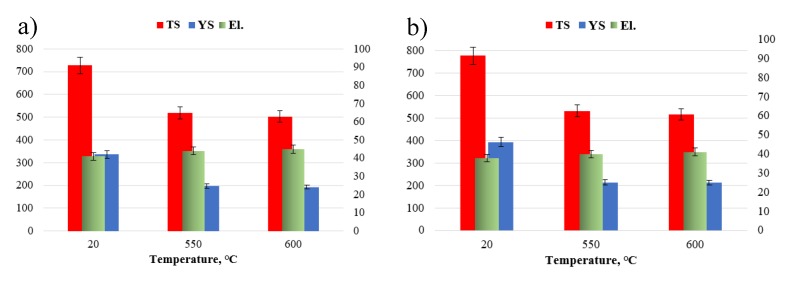
Mechanical properties of HR3C steel in as-received condition (**a**) and after service (**b**); YS—yield strength, TS—tensile strength, and El.—elongation.

In general, the purpose of the high-temperature solution treatment of the creep-resistant austenitic steels by dissolving the secondary phases was to obtain a single-phase structure. The structure of austenitic steels in the as-received condition (Figure 1a,b) provides high plasticity (and also ductility [3]) with the relatively low strength properties—the yield strength (YS) and tensile strength (TS) (Figure 9a). High plasticity of austenitic steels in the as-received condition results, among other things, from their solid solution strengthening and a large number of slip bands [3,4]. 

The total strength of austenitic steels is governed by different strengthening mechanisms: Mainly precipitation and also solid solution strengthening [9,10]. The secondary phase precipitation processes (Figure 5 and Figure 6) that take place during the service lead to a considerable increase in precipitation strengthening of the creep-resistant austenitic steels. The strength properties, hardness, and creep properties of the steel are known to depend mainly on the size, number, and volume fraction of the secondary particles. Small size and great number of the precipitated phase cause a significant growth of precipitation hardening. In the precipitation strengthening of steels, two mechanisms of interaction between the dislocations and the secondary phase particles can be observed: The Orowan mechanism and the cutting mechanism. In the creep-resistant steels, the dominant mechanism of interaction between the dislocations and the precipitates, causing the strengthening of these alloys, is the Orowan mechanism, because the precipitates in this group of materials are regarded as too hard to be cut by dislocations [4,11]. In the test steel after service, the value of the TS, regardless of the test temperature, was higher than the required one, while maintaining the plasticity (elongation (El.)) above the minimum value (Figure 9). In the test steel after service, two types of secondary precipitates were observed: M_23_C_6_ carbides and Z-phase (Figure 5 and Figure 6), therefore, the high strength properties should be associated with the occurrence of the precipitation strengthening by these precipitates. The dispersive form of the secondary precipitates of the Z-phase (Figure 6 and Figure 8) and the around 10-fold higher stress of the pinning force of this phase, compared to that of the M_23_C_6_ carbides, according to [11,24], is responsible in 75% for the effect of precipitation strengthening in austenitic steel. A similar effect was observed in P91 steel, where the stress required to bypass the primary NbC precipitate by the Orowan mechanism amounted to 16 MPa, whereas for the secondary VX precipitate—106 MPa, and for the M_23_C_6_ carbide—39 MPa [25]. Compared to M_23_C_6_ carbides, the Z-phase precipitates are characterised by a relatively more dispersive form and higher stability [9,10]. This makes them more effective barriers to the dislocation movement, assuming the Orowan mechanism effect. Moreover, part of the Z-phase particles are precipitated at the dislocations, which results in the pile-up of the dislocations—Figure 8. A similar favourable influence of the dispersive secondary precipitates on the considerable growth of YS in Super 304H steel, or TP 347 steel, was observed respectively in [26,27] and [28]. However, the significant influence of M_23_C_6_ carbides on YS at elevated temperature was pointed out by the authors of [29]. At the same time, during service at elevated temperatures, a gradual decrease in the concentration of solute elements occurs in the austenitic matrix [30], which translates into a reduction in effect of solid solution strengthening mechanism on the strength properties of the steel. This indicates that the increase in strength—YS and TS of the steel is mainly controlled by the precipitation strengthening mechanism. This shows that the dominant factor that affects the growth of strengthening translating into the increase in YS in HR3C steel is not only the presence of precipitates of the secondary Z-phase, but probably also of the M_23_C_6_ carbides. The strengthening effect associated with the Z-phase particles, and also probably with the M_23_C_6_ carbides, prevents the softening of the steel due to depletion of solid solution. The extent of strengthening will depend on the dispersion of these precipitates—the number, size, and shape. Trotter and Baker [31] show that the YS is controlled by the interparticle spacing in the grain interior. The increase in strength properties of the test steel during service results in a relatively slight reduction in yield strength–El. compared to the as-received condition (Figure 9). The reduction in El. is probably related to both the effects of the secondary precipitates/dislocations and to the likely decrease of twins density. According to [7,21], one of the effects of the influence of temperature on the microstructure of austenitic steels is the loss of twins. In the opinion of [32], the relatively high El. of HR3C steel after service may result from the grain rotation during deformation, which is probably related to the formation of the chromium-depleted area adjacent to the grain boundaries. 

The rise in test temperature results in the reduction in TS value by almost half compared to the as-received condition (Figure 9). Wang et al. [33] reported that at 650 °C fast diffusion could assist dislocation in bypassing nanosized precipitates by means of climbing. With the decreasing interaction between the precipitates and the matrix, the precipitate strengthening gradually weakened and the tensile strength subsequently decreased. Xiao et al. [34] also reported that this phenomenon might be related to sub-grain coarsening and decreasing in dislocation density.

**Figure 10:**
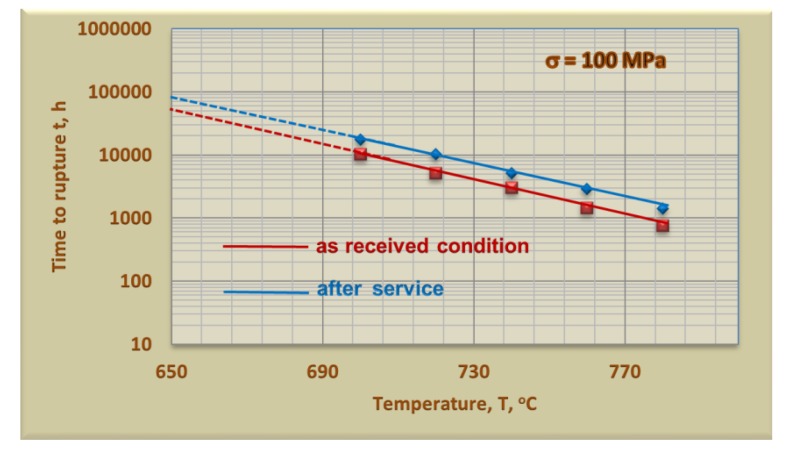
Results of short-term creep test of HR3C steel.

The factor deciding about the fitness of the material used, among other things, for the steam pipelines is the resistance to creep. The results of short-term creep tests of the test steel are presented in Figure 10. In the engineering practice, short-term creep tests are used to determine the time of further safe operation of the steel types serviced beyond the design service time [8]. The determination of residual life by the short-term creep tests provides knowledge about the actual possibility of further safe service of the given material and allows the time of further safe operation to be determined.

The creep strength of the elements of power units made of HR3C steel determined in the short-term creep tests, while maintaining the previous parameters, was higher than that of the material in the as-received condition. This effect, similarly, as in the case of the determined strength properties, was related to the occurrence of precipitation strengthening. In case of creep properties of the HR3C steel, it is determined by the dispersion and stability of the dominant precipitates in this alloy, i.e., the Z-phase particles, as well as the M_23_C_6_ carbides. The dispersive form of the Z-phase particles often precipitates at the dislocations [9,10,11,23,28], which enables their pinning, and their high thermodynamic stability [9,10] ensures the effective impeding of the free dislocation movement for a long time. Observed in the microstructure, the relatively fine M_23_C_6_ precipitates at the grain boundaries probably also have an influence on the increase in the creep rate limiting through the slide on the boundaries of grains. However, at elevated/high temperature, the mobility of dislocations grows and they can bypass the precipitates through climbing and cross slip, which limits the precipitation strengthening by Orowan mechanism. In addition to the precipitation hardening, an important factor that affects the creep properties of austenitic stainless steel is, according to [20], the solid solution strengthening by nitrogen atoms. 

## 5. Summary

The service of HR3C steel at the temperature lower than the long-term service temperature expected for this grade contributed to changes in the microstructure, reflected mostly in the precipitation processes. In the microstructure, the precipitation processes were observed at the grain boundaries and inside the grains. However, no precipitates at the twin boundaries were revealed. The precipitates at the grain boundaries formed the so-called continuous grid of precipitates. The presence of numerous chromium-rich precipitates in the microstructure of the test steel, the M_23_C_6_ carbides at the grain boundaries and the dispersive Z-phase precipitates inside the grains was observed. In the test steel, the precipitation processes resulted in the growth of strength properties and creep resistance through the precipitation strengthening mechanism. The effect of this strengthening mechanism depends on the stability and dispersion of the secondary precipitates. Controlling the precipitations and growth of the Z-phase and M_23_C_6_ carbides is beneficial to the enhancement of the strength and creep properties. 

## Figures and Tables

**Table 1:** Chemical composition of the test steel, %mass

C	Si	Mn	P	S	Cr	Ni	Nb	N	C
0.06	0.43	1.19	0.015	0.010	25.10	19.70	0.42	0.22	0.06
